# Cytotoxic Effect In Vitro of *Acalypha monostachya* Extracts over Human Tumor Cell Lines

**DOI:** 10.3390/plants10112326

**Published:** 2021-10-28

**Authors:** Gloria A. Guillén-Meléndez, Sheila A. Villa-Cedillo, Raymundo A. Pérez-Hernández, Uziel Castillo-Velázquez, Daniel Salas-Treviño, Odila Saucedo-Cárdenas, Roberto Montes-de-Oca-Luna, Christian A. Gómez-Tristán, Aimé Jazmín Garza-Arredondo, Diana Elisa Zamora-Ávila, María de Jesús Loera-Arias, Adolfo Soto-Domínguez

**Affiliations:** 1 Departamento de Histología, Facultad de Medicina, Universidad Autónoma de Nuevo León, Monterrey C.P. 64460, NL, Mexico; gloria.guillenmln@uanl.edu.mx (G.A.G.-M.); svilla.me0121@uanl.edu.mx (S.A.V.-C.); daniel.salastr@uanl.edu.mx (D.S.-T.); odila.saucedocr@uanl.edu.mx (O.S.-C.); robertomontesdeocaln@uanl.edu.mx (R.M.-d.-O.-L.); christian.gomeztrs@uanl.edu.mx (C.A.G.-T.); 2 Departamento de Química, Facultad de Ciencias Biológicas, Universidad Autónoma de Nuevo León, San Nicolás de los Garza C.P. 64455, NL, Mexico; raymundo.perezhrz@uanl.edu.mx; 3 Departamento de Inmunología, Facultad de Medicina Veterinaria, Universidad Autónoma de Nuevo León, Escobedo C.P. 66050, NL, Mexico; uziel.castillovl@uanl.edu.mx; 4 Departamento de Genética Molecular, Centro de Investigación Biomédica del Noreste (CIBIN) del IMSS, Monterrey C.P. 66720, NL, Mexico; 5 Departamento de Reproducción, Facultad de Medicina Veterinaria, Universidad Autónoma de Nuevo León, Escobedo C.P. 66050, NL, Mexico; aime.garzaarr@uanl.edu.mx; 6 Departamento de Genética, Facultad de Medicina Veterinaria, Universidad Autónoma de Nuevo León, Escobedo C.P. 66050, NL, Mexico; diana.zamoravl@uanl.edu.mx

**Keywords:** human tumor cell lines, cytotoxic effect, plant extracts, *Acalypha monostachya*

## Abstract

*Acalypha monostachya (A. monostachya)* is a plant that is used in traditional medicine as a cancer treatment; however, its effect has not been validated. In this study, the potential cytotoxic effects and morphological changes of *A. monostachya* were evaluated in human tumor cell lines. The aqueous (AE), methanolic (ME), and hexane (HE) extracts were obtained, and flavonoid-type phenolic compounds were detected, which indicates an antineoplastic effect. We observed a time-dependent and concentration-selective toxicity in human tumor cells. Additionally, the ME and HE showed the greatest cytotoxic effect at minimum concentrations compared to the AE, which showed this effect at the highest concentrations. All extracts induced significant morphological changes in tumor cells. The HeLa (cervix carcinoma) cells were more sensitive compared to the MDA-MB-231 (triple-negative breast cancer) cells. In conclusion, we demonstrated a cytotoxic in vitro effect of *A. monostachya* extracts in tumoral human cell lines. These results show the potential antineoplastic effects of *A. monostachya* in vitro. Hereafter, our lab team will continue working to usefully isolate and obtain the specific compounds of *A. monostachya* extracts with cytotoxic effects on tumor cells to find more alternatives for cancer treatment.

## 1. Introduction

According to the World Health Organization, cancer is defined as a process of uncontrolled cell growth and dissemination that can appear practically anywhere in the body [1]. In 2020, there were 19,292,789 cases of cancer presented around the world, of which 9,958,133 resulted deaths from this pathology [2]. In 2020 in Latin America and the Caribbean, the incidence of cancer in both sexes was 1,470,274 cases, and there was a mortality of 713, 414 [2]. In Mexico, there were 195,499 new cases and 90,222 deaths in 2020 [3]. Currently, there are multiple treatments for cancer depending on the type and stage of cancer. The most common methods are surgery, radiation therapy, and chemotherapy; other methods include immunotherapy, targeted therapy, hormone therapy, stem cell transplantation, and precision medicine [4].

For centuries, the knowledge surrounding the medicinal usage of plants has been passed down from generation to generation, and this knowledge has evolved based on observations, experience, and trial and error experiments. One of the advantages of the use of medicinal plants is their availability and the fact that they are culturally acceptable, as these plants are commonly used by indigenous populations because of these qualities [5].

*Acalypha* is the fourth largest genus in the *Euphorbiaceae* family, with approximately 450 to 570 species. Many of these species are used as medicinal plants, with their use occurring mainly in Africa and on the Mascarene Islands. The entire plant, including the leaves, stems, and roots, is commonly used in traditional remedies [6]. The leaves of the *Acalypha* species are succulent with sap stems, which tend to fall off with age. They are alternate, petiolate, or subsessile and have an entire sheet, crenate, or are toothed. The staminate flowers have four to eight stamens and vermiform anthers. The pistillate flowers are often prominently bracts with three sepals, three carpels, and one ovule per carpel and each divided style. Several species of *Acalypha* share the characteristic of allomorphic pistillate flowers and fruits [7].

Most *Acalypha* species are used as medicinal plants in West and East Africa, especially in Nigeria. Each part of the plant, including the leaf, stem, and roots, is used to make mixtures and decoctions to treat various ailments. *Acalypha* species such as *A. wilkesiana Müll. Arg., A. communis Müll. Arg.*, and *A. indica* L. are used in folk medicine as diuretics, anthelmintics, and for respiratory problems such as bronchitis, asthma, and pneumonia [8]. *A. wilkesiana*, *A. indica*, and *A. hispida Burm.f.* are common species found in Mauritius [9]. The Mauritian population uses *A. indica* leaves as well as whole plant as a treatment for skin infections such as scabies and dermatitis; *A. wilkesiana* is used to control diabetes, dysentery, and asthma. *A. integrifolia* is used as an astringent, purgative, and to eliminate intestinal worms as well as to cure various skin infections [9,10].

*Acalypha monostachya* (*A. monostachya*) is a perennial herb found in the southwestern United States and Mexico. It is used as a medicinal plant by the inhabitants of San Rafael and Zapotitlán Salinas, and Puebla, Mexico against skin eruptions, wounds, and diarrhea. In northern Mexico, it can be found in Bustamante, Nuevo León and, is commonly called the “Cancer Herb” and is used in the form of an infusion by boiling the leaves and inflorescences; it is also used in conjunction with *Bougainvillea glabra* for the treatment of colic and external tumors [11]. A study with a methanol extract (ME) of *A. monostachya* showed antimicrobial and antioxidant activities as well as toxicity against *A. salina* [12]. Another study evaluated the presence of micro and macronutrients in *A. monostachya* and concluded that it contains large amounts of Mg^+2^, Fe^+3,^ and Zn^+2^, so an analysis of its potential antioxidant effect is recommended [13,14]. This may also be confirmed by the fact that strong zinc accumulation has been detected in a closely related species (*A. alopecuroidea*) [15].

It has been observed by a phytochemical screening and through antimicrobial effect tests that the aqueous extract (AE) of *A. monostachya* contains carbohydrates and flavonoids as well as antimicrobial activity against *Pseudomonas aeruginosa* and *Staphylococcus aureus*. In the ethanolic extract (EE), positivity for flavonoids and terpenes and antimicrobial activity against the aforementioned bacteria and *Escherichia coli* were obtained [16].

Other studies have demonstrated the anticancer activity of the *Acalypha* genus, methanolic (ME), hexane (HE), and chloroform extracts of *A. indica*. These extracts were not cytotoxic to Vero (non-tumor kidney) cells and exhibited anticancer activity against NCIH187 (lung carcinoma) cells. Likewise, L-quebrachitol was purified from the extract, which was characterized by NMR [17].

A moderate cytotoxic effect of *A. fruticose* AE was demonstrated, which inhibited the proliferation of MDA-MB-435S (melanoma cell line) and Hep3B (hepatocellular carcinoma) cells in addition to the protection of DNA against oxidative damage induced with H_2_O_2_ [18]. *A. wilkesiana* exhibits an antiproliferative effect on U87MG (likely glioblastoma cells), A549 (lung carcinoma cells), and MCR5 (non-tumor lung) cells with their ethyl acetate extract, and a morphological study confirmed apoptosis and DNA damage [19]. The growth inhibition effect on MDA-MB-468 and MCR5 cells has been evaluated using the EE of *A. wilkesiana* [20]. 

Ethyl acetate extract (EE) combined with β, γ, and δ tocotrienols treatments of *A. wilkesiana* has potent antiproliferative effects on A549 and U87MG cells [21]. Studies indicate cytotoxic activity in HepG2 (hepatocellular carcinoma) and MCF7 (breast carcinoma) cells using EE and *A. wilkesiana* fractions showing 75.8% and 87.1% inhibition, respectively. Antioxidant activity was also observed by performing a DPPH test [22]. On the other hand, the anti-proliferative activity of the ME and fractions of *A. californica* were analyzed. Particularly, the hexanoic fraction inhibited RAW 264.7, HeLa, and L929 cells. This fraction was analyzed by molecular exclusion chromatography, obtaining terpenes and steroids, and its residual fraction contains tannins. Through HPLC, the presence of the compounds β-sitosterol and sigmasterol was demonstrated [23].

At this time, the use of medicinal plants as therapeutic agents is widely extended; *A. monostachya* is a plant that is used in traditional medicine as a cancer treatment; however, its effect has not been validated. For this reason, in the present study, we analyzed whether this plant has an in vitro antineoplastic effect on human tumor cell lines, specifically on two of the most frequent types of cancer that affect women in the world: breast cancer and cervical cancer.

## 2. Results

### 2.1. Taxonomic Identification of the Plant

The plant *A. monostachya* was identified as a perennial herb that is approximately 10 to 40 cm tall with numerous branching stems with blades that are the same length and width as the petioles (0.5 cm to 2.5 cm long). It has red male and female inflorescences on the same plant: the terminal male ones, and the axillary or terminal female ones.

### 2.2. Extract Yields and Phytochemical Screening

The yields obtained from the extracts AE, ME, and HE by the maceration method were 9.8%, 10.7%, and 2.2% *w*/*w*, respectively. All three extracts showed a green-brown color. Phytochemical analysis showed the presence of unsaturations, phenols, coumarins, lactones, flavonoids, saponins, aromatic compounds, carbohydrates, and carbonyl groups in the three extracts. Only ME and HE showed the presence of steroids and terpenoids. AE and ME were positive for sesquiterpene lactones and only AE showed alkaloids (Table 1).

The results obtained in the phytochemical analysis for each one of the extracts were compared with negative control, which was made up of the solvent used in each test, and subsequently, the corresponding reagents were added without the presence of any crude extract. Then, through a semi-quantitative analysis based on colorimetric and precipitation reactions, the presence of different groups of secondary metabolites was determined. The results were represented semi-quantitatively with crosses according to the intensity observed in each reaction (Table 1).

### 2.3. A. Monostachya Extracts Induce Morphological Changes in Cultured Human Tumor Cells

**Figure 1:**
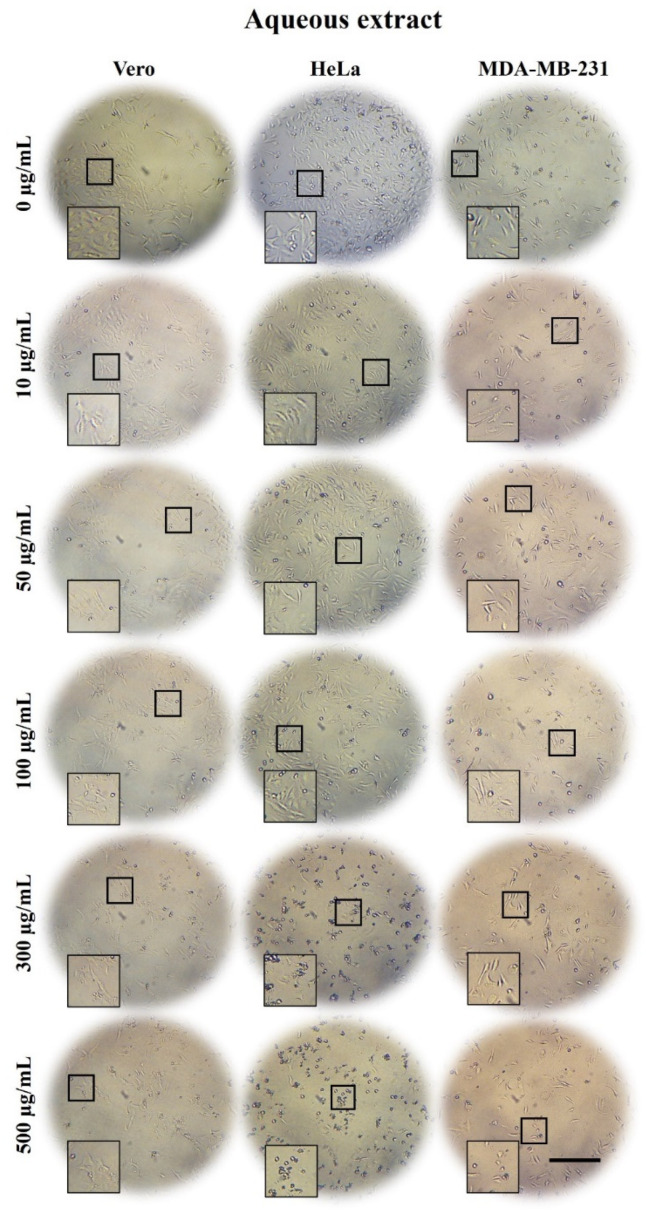
Micrographs of Vero, HeLa, and MDA-MB-231 cells exposed for 24 h to 0, 10, 50, 100, 300, and 500 µg/mL of *A. monostachya* AE. The tumor cells showed decrease confluence and morphological alterations such as rounding and loss of adhesion upon exposure to the treatments. These changes were accentuated at concentrations of 300 µg/mL and 500 µg/mL. HeLa cells showed greater changes compared to MDA-MB-231 and Vero cells, which showed minimal changes at this time point. These results were time- and concentration-dependent. Scale bar = 200 µm.

**Figure 2:**
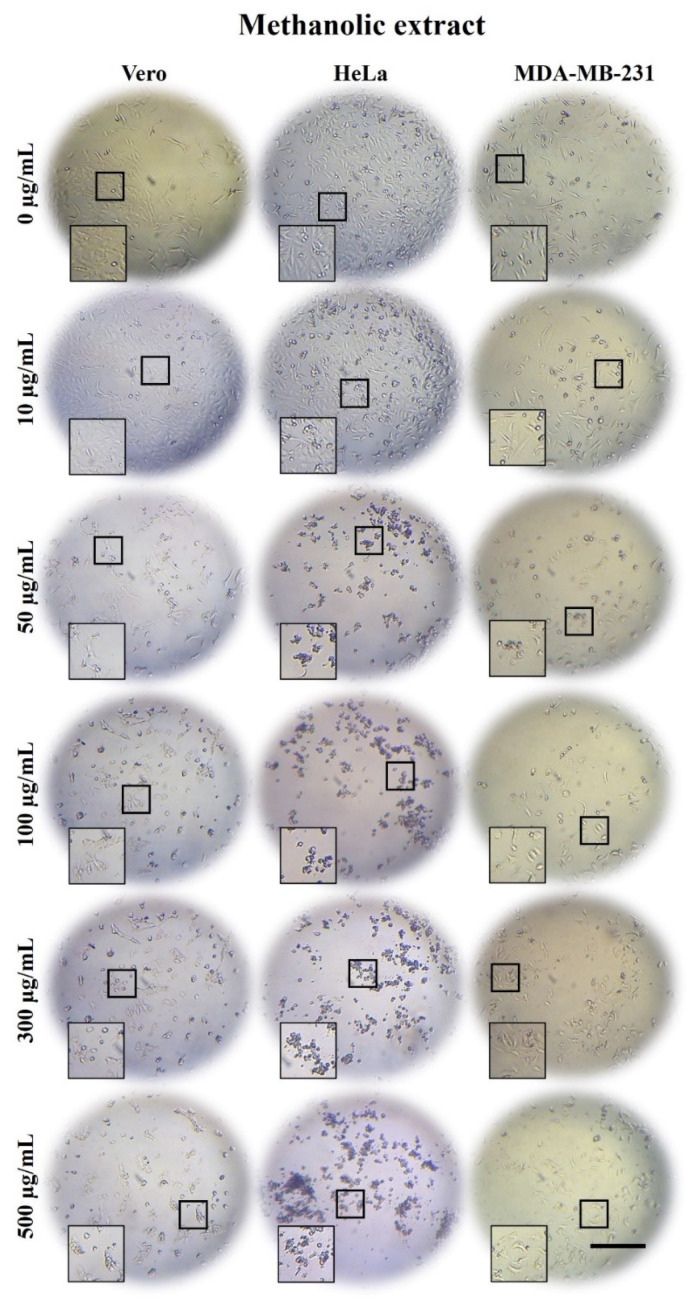
Micrographs of Vero, HeLa, and MDA-MB-231 cells exposed for 24 h to 0, 10, 50, 100, 300, and 500 µg/mL of *A. monostachya* ME. The tumor cells showed a marked decrease in confluence and morphological alterations as well as loss of adhesion after exposure to the extract. These changes were present from the concentrations of 50 µg/mL and higher. The HeLa cells showed more evident changes compared to Vero cells, which showed minimal changes, followed by the MDA-MB-231 cells. These alterations were time- and concentration-dependent. Scale bar = 200 µm.

**Figure 3:**
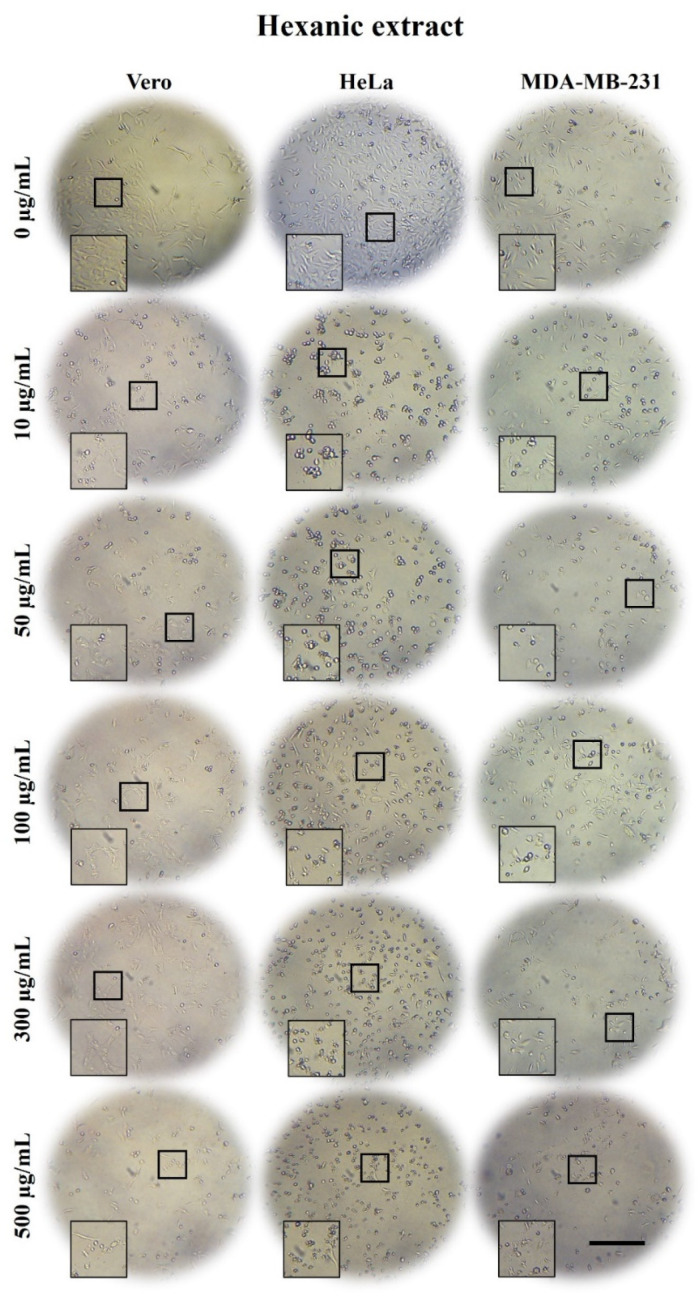
Micrographs of Vero, HeLa, and MDA-MB-231 cells exposed for 24 h to 0, 10, 50, 100, 300, and 500 µg/mL of *A. monostachya* HE. The tumor cells showed evident morphological alterations such as rounding and loss of adhesion as well as a marked decrease in confluence after exposure to the lowest concentration (10 µg/mL) of extract. The HeLa cells showed more evident changes vs. Vero cells, followed by the MDA-MB-231 cells, which showed these changes at higher concentrations. These alterations were time- and concentration-dependent. Scale bar = 200 µm.

To determine the cytotoxic effect of the extracts on tumor and non-tumor cells, light microscopy observations were performed after treatments at concentrations of 0, 10, 50, 50, 100, 300, and 500 µg/mL for 12, 24, 48, and 72 h. Figure 1, Figure 2 and Figure 3 show bright-field micrographs of Vero, HeLa, and MDA-MB-231 cell lines, respectively, after exposure to the concentrations of the extracts for 24 h.

Concentration-dependent changes were observed upon exposure to the three extracts, mainly in tumor cells; HeLa cells were the most affected after 24 h (Figure 1, Figure 2 and Figure 3) compared to MDA-MB-231. These changes were accentuated in HE-treated cells followed by those treated with ME and AE. On the other hand, reduced confluence was observed when tumor cells were treated for 12 h with the highest concentration (500 µg/mL), mainly with the HE, suggesting a cytotoxic and antiproliferative effect. Morphological changes were also observed as the cellular contraction, acquiring a rounded shape caused by the loss of adhesion. At 48 and 72 h, confluence decreased considerably, and morphological changes increased, suggesting a time-dependent effect.

These results were compared with those of the control cells, which were treated with vehicle; no such changes occurred in the control cells. In non-tumorigenic Vero cells, a slight decrease in confluency was observed at a concentration of 500 µg/mL after 24 h.

### 2.4. A. Monostachya Extracts Induce Changes in the Nuclear Morphology of Human Tumor Cells

**Figure 4:**
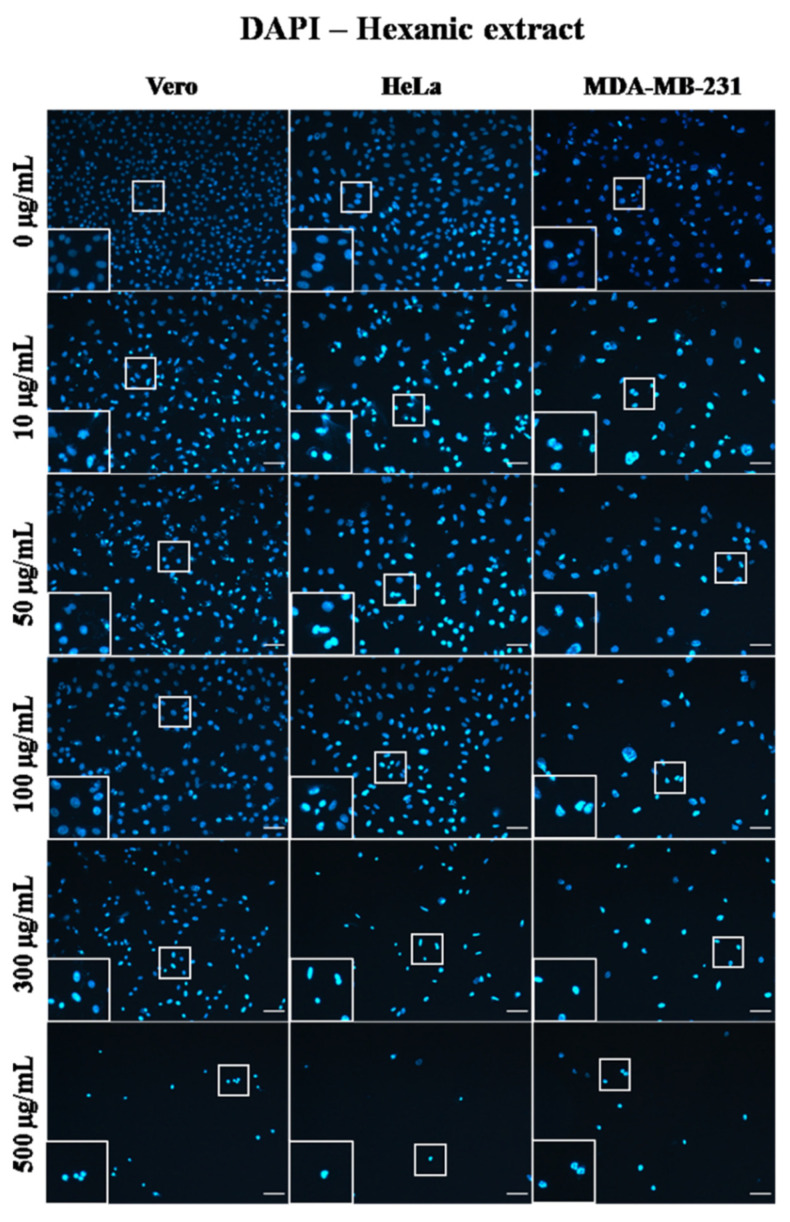
Micrographs of Vero, HeLa, and MDA-MB-231 cells exposed for 24 h to 0, 10, 50, 100, 300, and 500 µg/mL of *A. monostachya* HE. Nuclei labeling with DAPI. The nuclei of tumor cells showed obvious morphological alterations, such as pyknosis, rounding, and intense fluorescence as well as a marked decrease in confluence after exposure to the lowest concentration (10 µg/mL) of the extract. The HeLa cells showed more evident changes compared to Vero cells followed by the MDA-MB-231 cells, which showed these changes at higher concentrations. These alterations were also time- and concentration-dependent. Scale bar = 50 µm.

To evaluate confluence variations caused by the effect of the extracts, the cell adhesion area to the plate was quantified by a nuclei labeling assay with DAPI, proving the relationship between adherence and cell viability. At 12 h, the results were consistent with those observed by light microscopy. Tumor cells showed changes in nuclear morphology corresponding to cell death, described as chromatin condensation (karyolysis), rounding, decrease in size (pyknosis), and an intense fluorescence signal (Figure 4).

**Figure 5:**
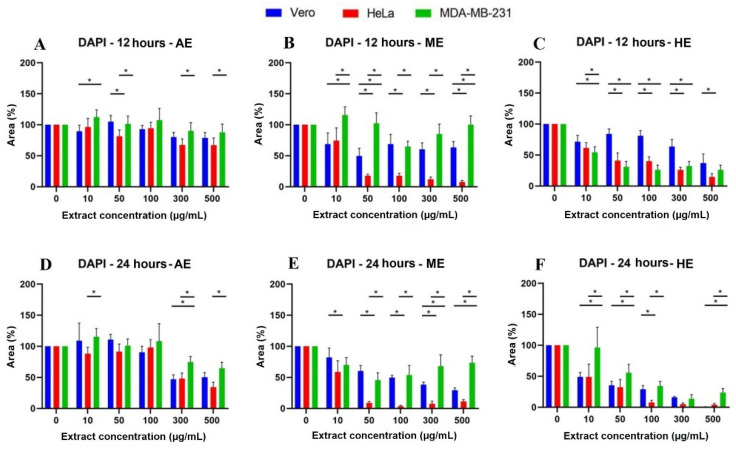
Quantitative analysis of nuclei labeling with DAPI in cells treated for 12 and 24 h with *A. monostachya* extracts. Cell area percentages are shown for Vero, HeLa, and MDA-MB-231 lines. HeLa and MDA-MB-231 lines exposed to concentrations of 0, 10, 50, 50, 100, 300, and 500 µg/mL of AE (*A. monostachya*) are shown for µg/mL of AE (**A**,**D**), ME (**B**,**E**), and HE (**C**,**F**). *n* = 8. * *p* < 0.05, Tukey’s multiple comparison test vs. Vero cells.

**Figure 6:**
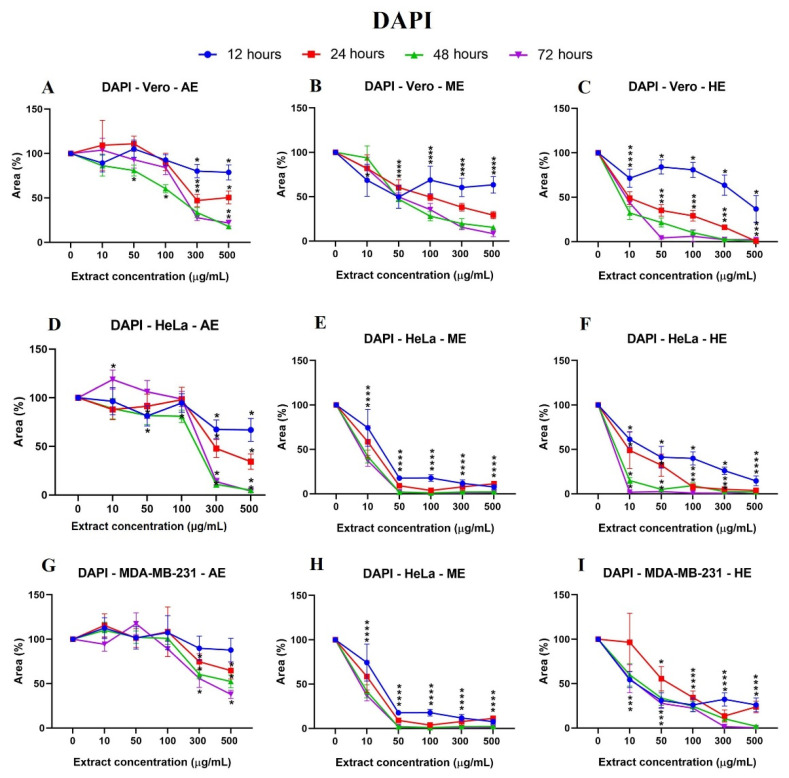
Cell behavior against different concentrations of *A. monostachya* extracts by nuclei labeling with DAPI. Comparisons of the effects of AE, ME, and HE on the cell lines: Vero (**A**–**C**), HeLa (**D**–**F**), and MDA-MB-231 (**G**–**I**) after exposure to the extracts at 12 h (blue line), 24 h (red line), 48 h (green line), and 72 h (purple line). The graphs show the % area as a function of the concentration of the extracts. It can be seen that HE has a greater cytotoxic effect on the tumor cell lines at 24 h and later. The HeLa cells showed a lower percentage of the area after exposure to the extracts. * *p* < 0.05, Tukey’s multiple comparison test vs. vehicle-treated cells.

Likewise, after calculating the percentage of the area, the results were represented in graphs showing the significant differences vs. Vero cells (Figure 5) and the behavior of the cells against the three extracts at the different times: 12, 24, 48, and 72 h (Figure 6). It was observed that HE significantly decreased the percentage of area at 24 h when using the concentrations of 300 and 500 µg/mL. In the case of HE and ME, this effect was observed at 24 h, starting at the 10 µg/mL concentration, and was accentuated in HE. Significant differences were observed vs. Vero cells, suggesting selective toxicity for tumoral cells.

### 2.5. A. Monostachya Extracts Decrease the Viability in Human Tumor Cells

**Figure 7:**
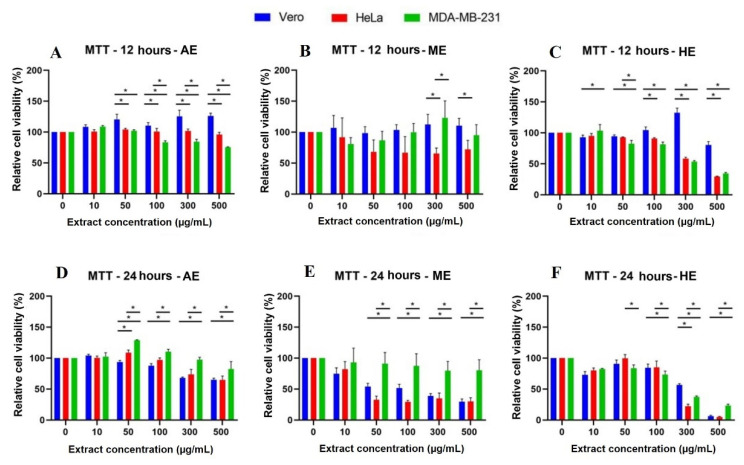
Cytotoxicity analysis by MTT assay in cells treated for 12 and 24 h with different concentrations of *A. monostachya* extracts. Relative cell viability percentages are shown for Vero, HeLa, and MDA-MB-231 lines. HeLa and MDA-MB-231 lines exposed to concentrations of 0, 10, 50, 50, 100, 300, and 500 µg/mL of AE (*A. monostachya*) are shown µg/mL of AE (**A**,**D**), ME (**B**,**E**), and HE (**C**,**F**). *n* = 8. * *p* < 0.05, Tukey’s multiple comparison test vs. Vero cells.

**Figure 8:**
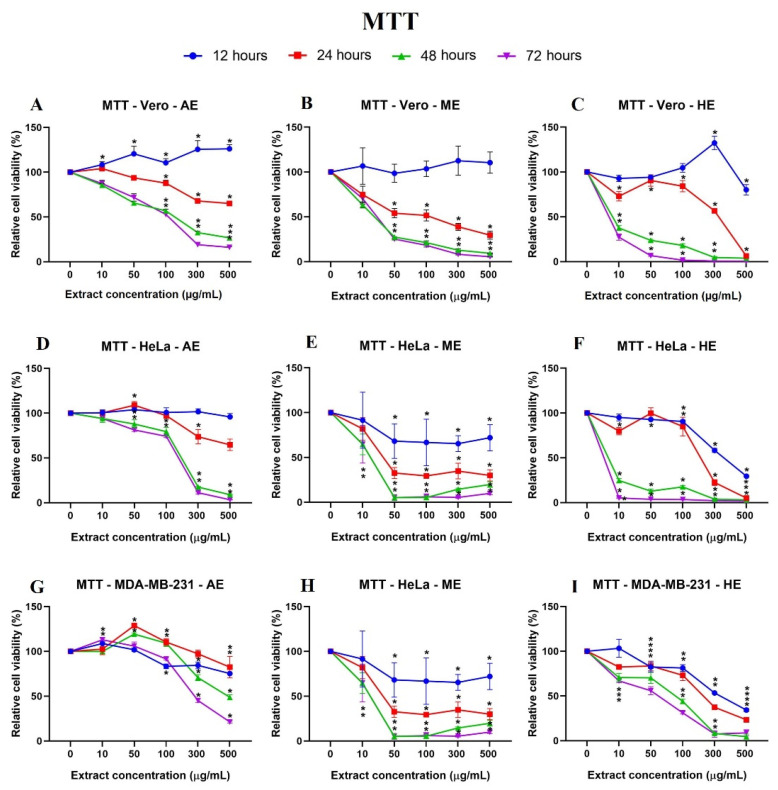
Cell behavior against different concentrations of *A. monostachya* extracts by MTT assay. Comparisons of the effects of AE, ME, and HE on the cell lines: Vero (**A**–**C**), HeLa (**D**–**F**), and MDA-MB-231 (**G**–**I**) after exposure to the extracts at 12 (blue line), 24 (red line), 48 (green line), and 72 h (purple line) are presented. The graphs show the % area as a function of the concentration of the extracts. It can be seen that HE has a greater cytotoxic effect on the tumor cell lines at 24 h and later. The HeLa cells showed greater sensitivity to the extracts. * *p* < 0.05, Tukey’s multiple comparison test vs. vehicle-treated cells.

In addition to quantifying viable cells by establishing a relationship with cell adhesion, we decided to perform an MTT assay to correlate mitochondrial activity with viability and thus observe cell energy metabolism behavior against the extracts. In this case, viable cells were able to metabolize the tetrazolium salt and reduce it to obtain the formazan, a purple-blue compound, whose absorbance readings allowed us obtain the following results: At 24 h, the results were similar to those observed at 12 h: AE showed significant differences against the control cell line when treated with the concentration of 300 µg/mL, and interestingly, ME and HE showed a cytotoxic effect from the concentration of 50 µg/mL onwards. At this exposure time, a concentration-dependent response began to be observed. In contrast, MDA-MB-231 cells showed resistance and, in some cases, higher relative viability than the Vero cell line (Figure 7). The longer the exposure time (48 and 72 h) to the three extracts, the more decreased the relative viability was (Figure 8).

The behavior of the cells with the extracts was compared as a function of time, and it should be noted that the HeLa cells, after 24 h, presented a lower percentage of relative viability after exposure to AE, ME, and HE. The MDA-MB-231 cells showed irregular behavior at 12 and 24 h. These results indicate a selective time- and concentration-dependent cytotoxic effect (Figure 6).

### 2.6. Human Tumor Cells Show More Morphological and Nuclear Alterations upon Exposure to A. Monostachya Extracts

After evaluating the morphological changes by light microscopy, fluorescence microscopy, and cytotoxicity by MTT assay, a morphological analysis was performed on semi-thin sections. For this purpose, the effect was evaluated at the concentrations at which the major morphological and viability changes were observed in the previous assays: for AE 300 and 500 µg/mL, and for ME and HE, the 10 and 50 µg/mL concentrations were used, respectively.

**Figure 9:**
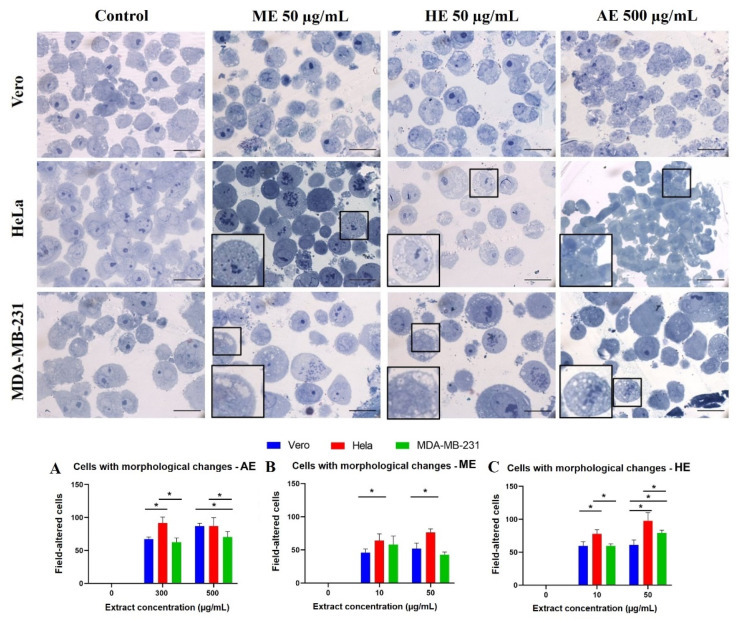
Evaluation of morphological changes in semi-thin sections stained with toluidine blue obtained from cells treated for 24 h with *A. monostachya* extracts. Note that upon exposure to the extracts, changes in nuclear morphology (condensation and displacement of chromatin towards the periphery), and cytoplasmic morphology (accumulation of vacuoles) were observed. Alterations in cell size were also observed, especially in tumor cells treated with HE. The HeLa cells showed more evident changes vs. the Vero cells followed by the MDA-MB-231 cells. The amplifications show changes in nuclear morphology and cytoplasm. Scale bar = 20 µm. Quantization of cells with morphological alterations related to cell death after exposure to *A. monostachya* extracts. Vero (blue bar), HeLa (red bar), and MDA-MB-231 (green bar) both after exposure to concentrations of 0, 10, 50, 300, and 500 µg/mL of AE (**A**), ME (**B**), and HE (**C**). *n* = 8. * *p* < 0.05, Tukey’s multiple comparison test vs. Vero cells.

First, the morphological characteristics of the untreated cells were evaluated, the Vero cells showed heterochromatic nuclei, a prominent nucleolus, and homogeneous cytoplasm. The tumor cells presented similar characteristics, but multiple mitotic events were observed, such as larger cellular and nuclear size compared to control, some presented two nucleoli. Moreover, the treated cells, especially tumor cells, showed vacuolizations in the cytoplasm when exposed to the lowest concentration that was tested (50 µg/mL and 300 µg/mL) of the three extracts (Figure 9).

Effects such as chromatin condensation were accentuated at the highest concentration were also observed and tended to be concentrated at the periphery of the nucleus, indicating that these results also occurred in a concentration-dependent manner. Interestingly, tumor cells treated with 50 and 500 µg/mL of the three extracts showed greater morphological alterations. Subsequently, cells showing morphological changes indicative of cell death were quantified. The results were in agreement with previous assays where it was observed that the HeLa cells presented significant differences vs. the control as well as a higher number of cells with morphological changes related to cell death compared to MDA-MB-231 and Vero cells (Figure 9).

## 3. Discussion

Plant secondary metabolites are chemical compounds produced by the plant cell through metabolic pathways derived from primary metabolism. They have been shown to have biological effects that provide the basis for traditional medicine in ancient communities [24]. In the results obtained in this study, the presence of flavonoid-type phenolic compounds stands out, which coincides with previous studies that also detected phenolic compounds in the ME of *A. monostachya*, from which benzoic acids, flavones, and flavonols were isolated. In addition, unsaturated fatty acids (linolenic and linoleic acid) were isolated from the HE [12]. These compounds are related to antimicrobial and antioxidant activity, and they are also possibly related to the cytotoxic activity demonstrated in our study.

Concerning the other *Acalypha* species, other types of compounds were also detected in the AE and ME, which are obtained from the leaves, stems, and roots of *A manniana*, *A. hispida, A. racemosa, A. marginata, A. indica*, and *A. alnifolia* that correspond to alkaloids, phenols, flavonoids, hydroxyanthraquinones, saponins, terpenes, tannins, and steroids, highlighting the presence of flavonoids in the whole plant [25,26,27,28]. These results coincide with our study by detecting groups of similar compounds in *A. monostachya*. The hexane fraction obtained from a crude ME of *A. hispida* showed the presence of flavonoids, carbohydrates, anthraquinones, cardiac glycosides, proteins, alkaloids, and the absence of tannins, sterols, and saponins [29]. In contrast, in our study, saponins were detected in low quanties in *A. monostachya*. In this regard, variations of secondary metabolites that occur between each species or even between the same species were found and are due to various factors, including the season, age, phenological state of the plant, water status, amount of nutrients, geographical location, and even the conditions in which the plant was collected [30]. These factors could explain the differences in the metabolites detected in our study.

Moreover, it has been shown that *A. indica* contains non-polar compounds with anti-inflammatory and anticancer activity in the HE and ethyl acetate extract showing the powerful inhibition of lipoxygenases and cyclooxygenases [31]; these compounds may be related to the high cytotoxic activity detected in the HE from *A. monostachya*. Likewise, the ME of *A. indica* contains L-Quebrachitol, a compound used for the synthesis of anticancer drugs [15], which may also be related to the activity of the ME in our study.

The morphological changes observed with light microscopy after the treatments that were previously described in response to the exposure of plant extracts [32]. These alterations can be described as membrane contraction, rounding, loss of contact with neighboring cells, the formation of membrane blisters, and apoptotic bodies, which are related to cell death due to apoptosis [32]. This indicates that, in this study, the observed cellular response is mainly attributed to the phenomenon of cellular death induced by the extracts of *A. monostachya*; however, more studies are required to confirm our results. In this aspect, the MTT assay is one of the most widely used methods to analyze cell proliferation and viability; it is absorbed by endocytosis and reduced by mitochondrial enzymes and endosomal/lysosomal compartments in order to be transported to cell surfaces, forming needle-shaped crystals of formazan. It has been previously reported that MTT does not cause injury or induce cell death [33]. The results of our study correlate with previous studies describing that the crude HE of *A. indica* has an IC50 of 50 µg/mL, and cytotoxic effects were observed from the concentration of 10 µg/mL to 100 µg/mL, where a slight increase in viability was observed [34]. 

Previous reports show that the *Acalypha* extracts obtained from non-polar solvents exhibited the highest antiproliferative activity and selective toxicity toward tumor cell lines [35] as well as the extracts of *A. monostachya*. This also suggests that the bioactive compound(s) of *Acalypha* are chemically non-polar compounds. One of the typical characteristics of cell death due to apoptosis is chromatin shrinkage and condensation, which was appreciated in our analysis with DAPI staining. Furthermore, it is reported that apoptotic cells present nuclear condensation and chromatin cleavage upon exposure to plant extracts [36]. These results were also observed in our investigation, with the DAPI nuclear staining of the tumor cells being exposed to the three extracts of *A. monostachya*. These results also suggest that the type of cell death that is induced is oriented towards apoptosis, according to our observations.

As mentioned above, among the morphological changes found by these extracts are those related to apoptosis, such as DNA fragmentation. Among the possible mechanisms of action that are proposed, it has been found that *A. wilkesiana* must simultaneously activate different mechanisms that cause single-stranded and double-stranded DNA breaks that lead to apoptosis. The constituents of the extracts can trigger different apoptotic pathways in different cancer phenotypes and can be specific to the cancer cell [19]. In accordance with these reports, we considered that the components of our extracts could also be inducing apoptosis by inducing DNA damage, as observed in the DAPI nuclei contrast assay; nevertheless, we need further molecular analysis to verify these findings.

On the other hand, the ME, HE, and chloroformic extracts of *A. indica* were not cytotoxic for Vero cells, but they were toxic for the tumor NCIH-187 cell line [17]; this agrees with our results, where we observed that the ME and HE of *A. monostachya* did not show a cytotoxic effect on Vero cells either. Interestingly, an antiproliferative effect of the AE of *A. fruticosa* on MDA-MB-435s cells has been reported [18]. In our study, this effect was not observed for *A. monostachya* AE. This could be due to the differences between species of the same genus, *Acalypha*.

Furthermore, cell morphology changes when the homeostasis conditions of the cells are affected by different processes, including cell death. These morphological alterations involve both the nucleus and the cytoplasm and are similar in all types of cells and species [37]. The main characteristics of apoptosis are the condensation of chromatin and nuclear fragmentation; the condensation forms a structure in the shape of a crescent or ring [38], which was observed in the semi-fine sections and with the DAPI stain. A characteristic of cell death due to apoptosis occurs when cells lose contact with neighboring cells and begin to form bulges on the plasma membrane known as bullae; the cells shrink, and finally, the bullae become well-known apoptotic bodies [39]. These characteristics were appreciated using light microscopy in a concentration-dependent manner.

Furthermore, necrosis differs in that it presents various morphological characteristics, such as dilation of the organelles, and in some cases, chromatin condensation and inflammation occur. In the case of autophagic and non-lysosomal cell death, the first is characterized by numerous vacuoles in the cytoplasm being filled with cellular debris, and the second shows the dilatation of organelles and empty spaces. Unlike apoptosis, the cell membrane becomes permeable very early on [40,41,42]. Interestingly, the results observed in the semi-fine sections indicate that the tumor cells treated with the extracts of *A. monostachya* have characteristics of both types of cell death, apoptosis, and necrosis.

There are also mitochondrial alterations that are described as markers of early apoptosis. When the permeability of the inner mitochondrial membrane is lost, fluid enters causing dilation, which has been observed to be similar to vacuolization under light microscopy [43]. The foregoing tests indicate that based on the observations of the alterations in nuclear morphology in greater detail and to know the composition of the vacuoles observed in our study, the evaluatation of these changes using transmission electron microscopy is suggested. There are other types of non-apoptotic cell death, which have been recently described that also involve the formation of cytoplasmic vacuoles, which are related to exposure to ischemic injury, cytotoxic compounds, or pathogens; these are paraptosis, oncosis, and methuosis [44]. Its characteristics include inflammation and cytoplasmic vacuolization. Taken together, the evidence described above suggests that it is necessary to perform tests that indicate the type of cell death that takes place in response to exposure to the extracts.

The MDA-MB-231 cell line showed sensitivity to phenanthrene derivatives isolated from *Juncus gerardii* [45]. The cytotoxicity observed and the resistance of the MDA-MB-231 cells to extracts of *A. monostachya* in our study have been observed previously when exposing the same cell line to extracts of *Origanum majorana* L.; there is an expression of survivin, which is a therapeutic target against breast cancer [46], which could explain the behavior that was observed by the cell line against extracts of *A. monostachya*. Together, these results provide information that is needed to continue the study of *A. monostachya* extracts. Our results demonstrated that *A. monostachya* extracts have a cytotoxic and morphological effect that is evidenced by nuclear and cytoplasmic morphological changes.

## 4. Materials and Methods

### 4.1. Plant Material

The aerial part (stems, leaves, and inflorescences) of *A. monostachya* were used, which werecollected from the Loma Larga Oriente in the municipality of San Pedro Garza García, Nuevo León (25°39′21.9″ N, −100°20′07.3.14″ W) in November 2020. The plant was identified and authenticated by Dr. Marco A. Guzmán-Lucio in the Facultad de Ciencias Biológicas, UANL. A specimen was deposited at the herbarium of this Faculty with accession number 030641.

### 4.2. A. Monostachya Extracts

The plant material was dried in the shade at room temperature (RT) for 3 days and was then ground using a manual grain mill (Victoria). The extraction of the plant material was performed by maceration at RT (23 ± 2 °C) with constant stirring at 200 RPM for 24 h using 20 g of plant and 400 mL of solvents (distilled water, absolute methanol, and n-hexane). The ME and HE were filtered and concentrated under reduced pressure (150 mbar) in a rotary evaporator (Yamato Scientific CO. LTD. RE-200) at 45 °C for 35 min.

To obtain the AE, an infusion was made with 20 g of the ground plant and 400 mL of distilled water at 95 °C and was stirred at 200 RPM for 1 h. After this time, the infusion was filtered and sterilized with a Corning^®^ syringe filter with a pore size of 0.22 µm and was subsequently subjected to evaporation in an incubator for 3 days at 40 °C. Once the solvent had been completely removed, the extract yield was obtained using the following formula:

(1) $$\mathrm{Yield}\;\left(\%p/p\right)=\frac{\mathrm{WE}}{\mathrm{IW}}\;\times \;100$$

where: 

WE: Weight of the extract obtained.

IW: Initial weight of the plant material.

Finally, the extracts were stored in glass flasks and were protected from light at 4 °C.

### 4.3. Partial Characterization (Partial Phytochemical Screening)

One of the first stages in the investigation of medicinal plants is the realization of a partial characterization, also called phytochemical screening, which consists of mixing the plant material with suitable solvents for the extraction and qualitative determination of the compounds present in the plant, which are mainly from the group of secondary metabolites. This is to provide the information that guides the research of the fractionation and/or isolation of bioactive compounds [47].

For this, colorimetric and precipitation reactions were performed. The results were semi-quantitative and were represented by crosses relating the intensity of the observed reaction with the presence of the plant compound: (−) not detected; (+) slightly positive reaction; (++) positive reaction; and (+++) strong positive reaction.

#### 4.3.1. Instaurations (KMnO_4_ Test)

An amount of 2 mg of the extracts was dissolved in 2 mL of water, acetone, or methanol, and three drops of 2% KMnO_4_ in water were added. The test was considered positive when discoloration or formation of a brown precipitate was observed, a result of the formation of MnO_2_.

#### 4.3.2. Carbonyl Group (2-4 Dinitrophenylhydrazine Test)

A 5 mg sample of the extracts was dissolved in 2 mL of ethanol, and 1 mL of a saturated solution of 2-4 dinitrophenylhydrazine in 6N HCl was added. The formation of a yellow or orange precipitate indicated the presence of the carbonyl group.

#### 4.3.3. Phenolic Compounds (Vegetable Tannins) (FeCl_3_ Test)

An amount of 2 mg of the extracts was dissolved in 2 mL of water or ethanol, and then three drops of 2.5% FeCl_3_ in water were added. The appearance of a red, blue-violet, or green precipitate was considered positive.

#### 4.3.4. Steroids and Terpenes (Salkowski Test)

An amount of 2 mg of each extract was dissolved in 2 mL of chloroform, and subsequently, 2 mL of H_2_SO_4_ was added. A positive result was considered for sterols and methyl sterols when a red-brown ring was formed at the interface.

#### 4.3.5. Carbohydrates

##### Molish Test

Molish’s reagent (1% alpha-naphthol in ethanol) was added dropwise to 2 mg of the extracts and then 2 mL of H_2_SO_4_ was added through the walls of the tube. The test was considered positive when a purple-colored ring formed at the interface.

##### Coumarin Test

An amount of 2 mg of the extracts was dissolved in 2 mL of ethanol and 10% NaOH was added dropwise. The test was considered positive if a yellow coloration was present and if it disappeared when the solution is acidified.

##### Lactone Test

An amount of 2 mg of the extracts was dissolved and 2 mL of an alcoholic solution of 10% NaOH was added. The test was considered positive when there was a turn to yellow or orange color that was lost or disappeared when a few drops of HCl were added, indicating the presence of a lactonic ring.

#### 4.3.6. Sesquiterpene Lactones (Baljet Test)

A amount of 2 mg of the extracts was dissolved in 2 mL of ethanol, and three drops of the mixed solution were added, with a positive result being indicated if it turned from orange to dark red. The 1:1 mixed solution consisted of Solution A, which contained 1% C_6_H_3_N_3_O_7_ in ethanol and Solution B, which contained 10% NaOH.

#### 4.3.7. Flavonoids (H_2_SO_4_ Test)

An amount of 2 mg of the extract was dissolved in 2 mL of H_2_SO_4_, and a positive result was indicated by yellow coloration for flavonoids, orange-cherry for flavones, red-bluish for chalcones, and red-purple for quinones.

#### 4.3.8. Alkaloids (Dragendorff Test)

The Munier and Macheboeuf modification [48] was used to determine the presence of alkaloids. An amount of 2 mf of the extract were dissolved in 2 mL of ethanol, and three drops of Dragendorff reagent were added. To prepare the reagent, two solutions were used: Solution A, which contained 0.85 g of Bi(NO_3_)_3_, which were mixed with 10 mL of CH₃COOH and 40 mL of water, and Solution B, which contained 8 g of KI dissolved in 20 mL of water. The reagent was prepared by mixing 5 mL of A, 4 mL of B, and 100 mL of water; the test was considered positive if a persistent red-orange coloration was present for 24 h.

#### 4.3.9. Saponins

##### NaHCO_3_ Test

The aqueous solution of 10% NaHCO₃ was prepared, and then 2 mg of the extracts were dissolved in 2 mL of water or ethanol, and three drops of concentrated H_2_SO_4_ were added. It was stirred slightly, and three drops of the NaHCO₃ solution were added. The appearance of bubbles and their permanence for more than 1 min indicated the presence of saponins.

##### Salkowski Test for Saponins

An amount of mg of the extract were dissolved in 2 mL of chloroform and 2 mL of H_2_SO_4_ was added thereto. The test was considered positive by the appearance of red color.

##### Aromaticity (H_2_SO_4_- CH_2_O Test)

A mixture of 1 mL of concentrated H_2_SO_4_ was prepared with a drop of CH_2_O (formaldehyde). An amount of 5 mg of the dissolved extract was dissolved in 1 mL of non-aromatic solvent (ethanol), and three drops of the above mixture were added, and when a red-violet color appeared, the test was considered positive.

### 4.4. Cell Lines

#### 4.4.1. Vero Cell Line

All cell lines were purchased from the American Type Culture Collection (ATCC) (Manassas VA, USA). Non-tumor Vero cells (ATCC: CCL-81) werederived from the kidney of an adult green monkey (*Cercopithecus aethiops*); they are adherent cells and with epithelial morphology. The cells were incubated in Advanced DMEM medium, 1× supplemented with 4% *v*/*v* of inactivated fetal bovine serum (FBS), 1% *v*/*v* of penicillin/streptomycin, and 1% of L-glutamine.

#### 4.4.2. HeLa Cell Line

Cells derived from adenocarcinoma of the human cervix (ATCC: CCL-2) from a 31-year-old Black female patient. They are adherent cells, with epithelial morphology and are positive for cytokeratin. They have been reported to contain human papillomavirus 18 (HPV-18) sequences [49]; additionally, these cells express low levels of p53 and normal levels of pRB [50]. The cells were incubated in 1X Advanced DMEM medium supplemented with 4% *v*/*v* of inactivated FBS, 1% penicillin/streptomycin, and 1% L-glutamine.

#### 4.4.3. MDA-MB-231 Cell Line

These cells were derived from human mammary adenocarcinoma (ATCC: CRM-HTB-26) from a 51-year-old Caucasian female patient. They are adherent cells of epithelial morphology and express the WNT7B oncogene. They express epidermal growth factor (EGF) and transforming growth factor-alpha (TGF α) [51]. Cells were incubated in 1× DMEM medium supplemented with 10% *v*/*v* of inactivated FBS, pyruvate 1×, 1% *v*/*v* penicillin/streptomycin, and 1% L-glutamine.

All cell lines were incubated at 37 °C in a 5% CO_2_ atmosphere.

### 4.5. Preparation of Working Solutions

From the crude extracts, stock solutions of 10 mg/mL were prepared to dissolve this concentration of each extract 50 µL of 100% dimethyl-sulfoxide (DMSO) and 950 µL of culture medium to obtain a DMSO concentration of 5%. Subsequently, solutions of 1 mg/mL were prepared to make a 1:10 dilution of the stock solution, with a DMSO concentration of 0.5%, this is the minimum non-toxic concentration [52]. Finally, from these solutions, the dilutions were prepared at concentrations of 0, 10, 50, 100, 300, and 500 µg/mL for the cell treatments.

### 4.6. Cytotoxicity Test with MTT(3-(4,5-Dimethylthiazol-2-yl)-2,5-Diphenyltetrazolium Bromide)

Mitochondrial function can be evaluated based on the activity of the reductases found in the organelle and are directly related to cell viability, and the MTT assay can be used to measure such activity. This compound is a yellow tetrazolium salt, which is reduced to formazan by the action of mitochondrial enzymes, especially succinate dehydrogenase, resulting in a purple-blue product that can be evaluated by spectrophotometry [53]. For this assay, 5 × 10^4^ cells per well (*n* = 7) were incubated for 24 h in a 96-well plate to allow adhesion. Subsequently, the treatments of each extract were applied at concentrations of 10, 50, 100, 300, and 500 µg/mL diluted in 100 µL of culture medium. Cell culture medium was used as a negative control (vehicle). The results were analyzed at 12, 24, 40, and 72 h. 

Two hours before the end of the corresponding incubation times, representative photomicrographs of each concentration were taken at 40× with a Southern Precision Instrument inverted microscope to observe the effect of the extracts on the cell morphology. Subsequently, 15 µL of MTT (3 mg/mL) were added to each well, and the cells were incubated for 2 h at 37 °C; then, the medium was removed, and the MTT developer reagent (4 mM HCl, 0.040% NP40 in isopropanol) was added. The plate was shaken at 125 RPM for 10 min, and an absorbance analysis was performed at 590 nm with a 620 nm reference filter in an iMark^TM^ microplate reader. The absorbance value is directly proportional to the number of metabolically active cells, which is an indirect measure of cell viability.

### 4.7. DAPI Nucleus Labeling Assay

To relate the results obtained in the MTT assay, the use of the fluorescent dye DAPI (4’,6-diamidino-2-phenylindole) that binds to the nucleic acids of adhered cells, allowed us to estimate the number of cells viable related to cell adhesion. For this, 96-well plate cultures were performed under the same conditions as MTT assay treatments (*n* = 8). At the end of the incubation times, the medium was removed, two washes of 200 µL were performed with 1 × PBS, and subsequently, the cells were fixed with methanol/acetone at a ratio of 1: 1 for 20 min at 4 °C. After the fixer was removed, two washes were performed again with 1X PBS, and 100 µL of DAPI (100 ng/mL) were added for 15 min at RT in the darkness. At the end of this period, the excess DAPI was removed, and the plate was dried and observed under fluorescence microscopy. Eight micrographs were taken per concentration at a total magnification of 100×, which were evaluated with ImageJ^®^ software version 1.51, (NIH) which calculated the percentage of the area covered by the adhered cells in each of the wells

### 4.8. Morphological Analysis

To evaluate the presence of morphological changes in the cells due to exposure to extracts of *A. monostachya*, 1 × 10^6^ cells were seeded in 60 mm dishes and were incubated for 24 h to allow adherence. Subsequently, the treatments of 0, 10, and 50 µg/mL of EM and EH and 0, 300, and 500 µg/mL of EA were applied and diluted in culture medium at a total of 3 mL per dish and were incubated for 24 and 48 h. At the end of the incubation time, the cells were harvested using 0.25% trypsin, two washes were performed with 1× PBS, and later, they were fixed with 2.5% glutaraldehyde buffered in 0.1 M cacodylate buffer (pH 7.4) for 24 h. Next, the cells were washed two times with cacodylate buffer for 5 min with centrifugation at 14,000 RMP; they were decanted, and a post-fixation was performed with OsO_4_ at 2% for 12 h; they were washed again and dehydrated with a gradient of ketones (30%, 50%, 70%, 90%, and 100%) for 5 min and embedded in middle epoxy resin for 72 h at 60 °C. From the epoxy blocks, 350 nm thick semi-fine sections were obtained by ultramicrotomy and subsequent staining with 1% toluidine blue for 1 min. One hundred cells were counted per section when analyzing the presence of changes in the nuclear morphology, chromatin condensation, and vacuolization in the cytoplasm to obtain the percentage of positive cells in each treatment.

### 4.9. Statistical Analysis

For the descriptive statistics, the quantitative variables were summarized by calculating the mean, and as a measure of dispersion, the standard deviation (SD). In the inferential statistics, the Kolmogorov–Smirnov normality test was applied to evaluate the distribution of the data, with all variables having a normal distribution. Subsequently, an ANOVA and a Tukey test were performed to evaluate the presence of the differences between the groups. All graphs, calculations, and statistical analyses were made using GraphPad Prism version 8.0 software for Windows (GraphPad Software, Inc., San Diego, CA, USA).

## 5. Conclusions

This research provides information on the effect of crude extracts of different polarities obtained from *A. monostachya* on human tumor lines, demonstrating that it possesses time- and concentration-dependent cytotoxic activity. This was evidenced by evaluating the relative viability and the presence of morphological alterations when the extracts were exposed. Likewise, this cytotoxicity is selective for tumor cell lines, highlighting that the HeLa cells presented more cytoplasmic and nuclear morphological changes as well as a lower percentage of relative viability when exposed to extracts compared to MDA-MB-231 cells, which responded in the same way to higher concentrations. This activity can be attributed to the phenolic compounds detected by the phytochemical screening being carried out.

The perspectives of this work are oriented to perform a fractionation guided by bioassay to obtain a fraction with the bioactive compound and thus isolate and purify it. With the data obtained, the IC50 of the crude extracts and subsequently their different fractions can be calculated. Likewise, it is convenient to evaluate the effect of the extracts in other tumor cell lines to compare the induced changes. Finally, it is possible to further evaluate the type of cell death to establish a mechanism by which the bioactive compounds present in *A. monostachya* act.

## Figures and Tables

**Table 1:** Partial phytochemical screening of the crude extracts of *A. monostachya*.

Phytoconstituents	Test	Observations	AE	ME	HE
Unsaturations	Potassium permanganate test	Brown precipitate	+++	++	+
Phenols	Ferric chloride test	Green color	++	+++	+
Terpenoids and steroids	Salkowski test	Reddish-brown ring formation	−	++	++
Coumarins and lactones	Sodium hydroxide test	Yellow color	++	++	+
Sesquiterpene lactones	Baljet’s test	Orange color	+	+	−
Flavonoids	Sulphuric acid test	Reddish color	+	+	++
Alkaloids	Dragendorff test	Orange precipitate	+	−	−
Saponins	Foam test	Presence of stable foam	+++	+	+
Carbohydrates	Molisch´s test	Purple ring formation	+++	++	+
Aromatic compounds	Formaline test	Red color	+	+	+
Carbonyl group	2-4 dinitrophenylhydrazine test	Orange color	++	+	+

− Not detected; + slightly positive reaction; ++ positive reaction; and +++ strong positive reaction. The first columns indicate the name of the secondary metabolite and the test to perform the detection. The next column shows the observations for each reaction followed by the results obtained in the extracts. Aqueous extract (AE), methanolic extract (EM), hexanoic extract (HE).

## Data Availability

The data and materials supporting the conclusions of this article are included within the article.
